# Adipsic hypernatremia in a young Sudanese child, challenges in a limited-resource setting: a case report

**DOI:** 10.11604/pamj.2021.38.86.26236

**Published:** 2021-01-26

**Authors:** Mohammed Abdulrahman Alhassan, Asmahan Tagelsir Abdalla, Samar Sabir Hassan, Mohamed Ahmed Abdullah

**Affiliations:** 1 Department of Pediatrics, Prince Sattam Bin Abdulaziz University, Alkharj, Kingdom of Saudi Arabia,; 2 Department of Pediatric Endocrinology, Dr. Gaafar Ibn Auf Children´s Tertiary Hospital, Khartoum, Sudan,; 3 Department of Pediatric Endocrinology, Faculty of Medicine, University of Khartoum, khartoum, Sudan

**Keywords:** Thirst, hypernatremia, hypothalamus, diabetes insipidus, child, case report

## Abstract

Adipsia is a rare condition characterized by a lack of thirst due to a defect in specific osmoreceptors located in the hypothalamus. The disorder is characterized by failure to maintain the body’s normal plasma osmolality (POSM), resulting in chronic or recurrent severe hypernatremia and dehydration. Adipsia is usually accompanied by central diabetes insipidus (DI). Isolated adipsia (without DI) is very rare, with causes ranging from congenital central nervous system malformations to acquired anterior hypothalamic lesions. The diagnosis and management of the condition are considerably challenging for both clinicians and patients/parents, especially in a resource-limited setting. We here in present the first case report of adipsia from Sudan; a young child with isolated adipsia, diagnosed after recurrent severe hypernatemic dehydration episodes. The report portrays the unique challenges in suspecting, diagnosing, and managing the condition in a limited-resource setting.

## Introduction

Adipsia is a rare condition characterized by a lack of thirst, with failure to maintain the body´s normal plasma osmolality (POSM), resulting in chronic or recurrent severe hypernatremia and dehydration. Thirst is triggered by osmoreceptors located in the anterior hypothalamus. These are adjacent to, but distinct from, similar osmoreceptors that stimulate arginine vasopressin (AVP) synthesis and secretion in response to increased POSM [1]. Both thirst and the AVP systems work in concert to keep POSM under tight control. The contiguous position of these osmoregulatory systems can explain why the vast majority of patients with adipsia are accompanied by central diabetes insipidus (DI) [2]. Reports of isolated adipsia (i.e. without DI) have been exceptionally rare, probably not beyond ten in the literature. The causes for isolated adipsia ranged from congenital central nervous system malformations, particularly those involving midline structures to acquired anterior hypothalamic lesions [3]. In this article, we are describing the first Sudanese child with adipsia. The adipsia in this patient is most likely isolated without DI. The child, in addition, has cleft lip and palate, microcephaly, spastic quadriparesis, epilepsy, and developmental delay.

## Patient and observation

A 20-month-old infant was referred to our pediatric endocrinology department at Gaafar Ibn Auf Children´s Hospital with suspected diabetes insipidus (DI) due to recurrent hospital admission with hypernatremia dehydration without gastrointestinal losses, and failure to thrive. She is the only child of healthy consanguineous parents. She was born prematurely at 36 weeks gestation by emergency caesarian section due to premature rupture of membranes (PROM). APGAR score was 9 and 10 at one and five minutes, respectively. Birth weight was low (2Kg). She was noticed at birth to have microcephaly, unilateral cleft lip, and cleft palate. The neonatal period was complicated by unconjugated hyperbilirubinemia and sepsis. At the age of two months, she developed focal then generalized seizures. After evaluation, a diagnosis of epilepsy was made, and she was prescribed oral phenobarbitone, then on sodium valproate, which was complicated by thrombocytopenia. She was finally shifted to levetiracetam and clonazepam with good control of the seizures and minimal side effects. At the age of six months, the cleft lip was repaired without intra/postoperative complications. She was fed by a special feeding teat for the cleft palate. The mother reported that her child was delayed in gross motor milestones and that she had become anorexic and very lazy with frequent attacks of fever with no focus since the age of 18 months. During routine evaluation in a remote health care facility, she was found to have hypernatremia. Although she was dehydrated, she did not seem to be thirsty, and this observation was augmented by the mother when she reported that her baby had never asked for water and was ''never thirsty''.

At admission to our department, she was irritable and dehydrated. She had microcephaly with a head circumference at -7 standard deviation score (SDS) (40cm), and weight and length at the 5^th^ and 50^th^ percentile, respectively. She had a repaired cleft lip, unrepaired cleft palate, depressed nasal bridge, and hypertelorism with no digital anomalies. Blood pressure was normal. Nothing else was remarkable on physical examination. She was resuscitated with intravenous fluids. Basic laboratory analysis revealed hypernatremia with high serum chloride and normal blood sugar (Table 1). Calculated serum osmolality was high at 373 mOsm/Kg with a high urine specific gravity (SG) at 1.030. Urine osmolality was not measured due to lack of availability. A 24-hour urine output was 400 ml/m2 (156 ml/24hours). Hypernatremia was slowly corrected at a rate of 6-8 mEq/L per day, with the fluid deficit replenished over around 48 hours using D5 ½ normal saline. Despite the disappearance of signs of dehydration, her serum sodium (Na^+^) level remained between 152-158 mmol/l (Table 2). During hospital admission, the child had never felt thirsty and never asked for water even during the dehydration attack. This observation was confirmed by the treating medical staff and persisted even after the patient developed clear adequate cognitive abilities to express her other physiological desires, such as hunger.

**Table 1:** basic biochemistry at 1^st^ admission

Date	S. Na^+^ mmol/l	S. K^+^ mmol/l	Serum Cl^-^ mmol/l	Blood sugar mmol/l	BUN mmol/l	Serum osmolality mOsm/Kg	Urine SG
Day1	173	4.5	142	4.7	22.5	373	1.030
Day2	156	4.7	122	5	2.82	320	1.025
Day3	154	4.2	82				
Day4	167	3.9	135				
Day5	152	4.1	128				

**Table 2:** changes of serum sodium (mmol/l) after inpatient intravenous fluid (IVF) therapy at different hospitalizations (hospital admissions usually took between 3-5 days)

Year	Pre IVF	Post IVF
2015	173	152
2016	186	156
2018	194	157
2019	180	158

Both computed tomography (CT) scan and magnetic resonance imaging (MRI) of the brain did not reveal any abnormalities in the hypothalamic/pituitary area. Further evaluation revealed normal anterior pituitary function, including thyroid tests, dynamic growth hormone test, serum cortisol, and adrenocorticotrophic hormone (ACTH) levels (Table 3). Direct measurement of serum AVP level was unavailable in our setting. The combination of high urine specific gravity and the absence of polyuria led us to put the diagnosis of adipic hypernatremia without DI. The patient was discharged home with the mother instructed to give her the obligatory oral fluids calculated as the maintenance plus the insensible losses, to be administered every 3 hours. She was also advised to increase the fluid volume during hot days. Since that time, she had kept dropping in the emergency room on and off with frequent attacks of mild fever, lethargy, oliguria, and hypernatremia dehydration. At the age of 5 years, she presented, in addition to microcephaly, with global developmental delay, spastic quadriparesis, and a recent tiptoe walking. She was seen by a neurologist who proposed the diagnosis of microcephaly-spastic tetraplegic cerebral palsy and started her on physiotherapy. Vision and hearing were normal. Seizures were controlled, and anti-epileptic drugs were tapered and stopped when she was six years old. Unfortunately, the patient succumbed to an acute illness before reaching the emergency department at the age of 6 and a half years.

**Table 3:** anterior pituitary hormones assessment

Test	Result (reference range)
Growth hormone (stimulated peak level)	14.5 ng/ml (>10)
Cortisol	643 nmol/L (138-635)
ACTH	7 pmol/L (2.2-13.3)
TSH	3.57 μIU/ml (0.27-5)
FT4	17.4 nmol/L (9-24)

## Discussion

We have described a young child with adipic hypernatremia, most likely without DI. While case reports of adipic DI are relatively visible in the literature, including a report from Africa [4], adipic hypernatremia due to a selective defect in the osmoregulation of thirst is extremely rare, particularly when it comes to probable congenital causes. In a recent case series, an autoimmune mechanism affecting relevant areas in the anterior hypothalamus in children has been proposed [5]. In our patient, younger age at onset, midline facial defect, and associated microcephaly suggests a developmental origin for the condition, despite a negative MRI finding. In our patient, the combination of high urine specific gravity and the absence of polyuria makes the co-existence of DI quite unlikely. Nevertheless, partial DI cannot be totally ruled out without a formal water deprivation test with urine osmolality testing, which was not done in our patient. Direct measurement of serum AVP level was unfortunately unavailable in our setting. The direct measurement of the serum hormone level, however, has well-known potential limitations. The absence of DI sheds yet another light on the discrete nature of the thirst osmoregulatory-system. The osmoreceptors of the thirst system appear to activate on slightly higher POSM levels than those of AVP. This guards against excessive drinking urges in humans.

Similar reports have defined adipsia without hypothalamic structural defects, with midline facial defects [6], with developmental delay [7], with other syndromic features [8], or without any other associated features [9]. This portrays a wide array of potential pathogenetic and molecular bases for the condition. In these cases, as well as in our patient, the reason for selective effectuation of the osmoregulatory stimuli for thirst is largely unexplained. Adjacent hypothalamic-pituitary axes in our patient appear intact, as evidenced by normal pituitary functions. A cleft lip and palate are indicative of a midline brain developmental defect, though this could not be appreciated in the MRI. A urine osmolality would have provided a firmer proof of the integrity of the AVP osmoregulation. Nevertheless, as mentioned earlier, the absence of DI in our case was logically based on the near oliguria and the high urine specific gravity. Our patient demonstrated the notoriously challenging nature of home fluid management in young children with adipsia. The addition of cleft palate, cognitive delay, and quadriparesis further complicated the situation. With increasing age, patients with adipsia may develop a social stimulus for drinking. Again, this was probably hindered by the cognitive defect in our patient. The hypovolemic thirst mechanism stimulates drinking through stretch receptors. The ameliorating or additive effect of this mechanism in our patient could not be elucidated. Adipsia confers a higher risk of infection and death than non-adipic DI [10].

## Conclusion

Adipsia in young children is rare and usually associated with a congenital central nervous system defect. History of lack of spontaneous drinking in the context of hypernatremia dehydration episodes is virtually diagnostic. Demonstration of normal to low output of appropriately concentrated urine will exclude the frequent co-occurrence of DI. Maintaining water balance in these children is challenging and requires careful calculation and possible enteral administration of daily fluid intake at home.
